# Physical and metabolic requirements of elite military divers

**DOI:** 10.3389/fphys.2025.1505363

**Published:** 2025-05-15

**Authors:** Karen R. Kelly, Laura J. Palombo, Andrea C. Givens, Jake R. Bernards, Daniel Bennett

**Affiliations:** ^1^ Warfighter Performance Department, Naval Health Research Center, San Diego, CA, United States; ^2^ Leidos, Inc., San Diego, CA, United States

**Keywords:** cortisol, testosterone, military, diving, caloric expenditure

## Abstract

**Introduction:**

The purpose of this investigation was to characterize the physical and physiological profile of elite military divers.

**Methods:**

The profile included anthropometric (height, weight, fat free mass, fat mass, percent body fat), performance testing ( $\dot{V}$ o_2_max, 3-mile run (4.82 km), 0.5-mile swim (0.8 km), weighted pull-ups, estimated 1-rep max for bench and deadlift, and broad jump) and physiological functioning via the awake response (cortisol, testosterone, and dehydroepiandrosterone).

**Results:**

Anthropometric and performance results presented as MEAN ± SE include: age: 28.0 ± 0.5 years; height: 70.7 ± 0.3 in (179.6 ± 0.8 cm); weight: 193.3 ± 2.0 lbs (87.9 ± 0.9 kg); body fat percentage: 18.2% ± 0.6%; $\dot{V}$ o_2_max: 55.3 ± 0.7 ml kg ^-1^ · min^-1^; bench-press 1RM: 278.7 ± 7.7 lbs (126.8 ± 3.5 kg); deadlift 1RM: 397.9 ± 10.6 lbs (172.7 ± 4.8 kg). Significant associations were found between anthropometric measures and measures of magnitude in testosterone and DHEA. Physical performance metrics showed significant associations with summary parameters in all salivary hormones, with quartile splits yielding significant differences in absolute DHEA and 1RM deadlift (*F* (3, 30) = 2.97, *p* = 0.048), AUCg testosterone and broad jump (*F* (3, 37) = 2.86, *p* = 0.05), and AUCg T:C ratio and 25lb weighted pull ups (*F* (3, 35) = 4.66, *p* = 0.008). Linear mixed models revealed a significant effects of evolution/collection time point on AUCg DHEA at time points three (B = −2735.96, *t* (177.32) = −2.39, *p* = 0.018) and four (B = −3089.92, *t* (178.97) = −2.7, *p* = 0.008); and on peak testosterone at time point five (B = 28.12, *t* (215.4) = 2.4, *p* = 0.017) with reference to time point one.

**Conclusion:**

The data presented herein indicate there are certain periods of training that elicit significant changes in testosterone and DHEA while cortisol remains stable throughout the training cycle. To our knowledge, this effort is the first to document changes in stress biomarkers over time in elite military divers.

## Introduction

The modern warfighter is exposed to extreme psychological and physiological stressors, prevalent not only in combat, but also during daily operational training (Nindl et al., 2007; Taylor et al., 2007; Castellani et al., 2017; Chapin et al., 2021; Hackney and Hodgdon, 1991). The physiological burden is even greater with physically demanding military occupations such as diving. Diving increases cardiovascular strain due to the pressure of the undersea environment, thereby increasing overall physiological stress (Castellani et al., 2017; Lundell et al., 2019; Marlinge et al., 2019; Schipke and Pelzer, 2001). Recent attention has focused on the effect of SCUBA diving on biochemical changes during recreational diving, which typically is of shorter duration than military diving (Zarezadeh and Azarbayjani, 2014; Weist et al., 2012). These efforts have focused on acute stress biomarkers as well as indicators of cardiovascular health and immune function and have demonstrated that short term repetitive diving significantly changes markers of stress, endothelial and cardiac damage, and promotes inflammation (Marlinge et al., 2019; Zarezadeh and Azarbayjani, 2014; Weist et al., 2012). The change in biomarkers was attributed to the combination of physiological effects of immersion and environmental pressure (Lundell et al., 2019; Earp et al., 2019; Sramek et al., 2000). Military divers regularly conduct dive training and operations as part of their annual training cycle, diving multiple times per week over long periods of time [6–9 months] which could lead to overtraining syndrome (OTS).

Overtraining syndrome is a widely accepted term that characterizes a range of physiological maladaptations that can lead to performance reductions, changes in behavior and mood, and increased risk of injury/illness (Armstrong et al., 2021). Chronic exposure to multifactorial stress, such as with military diving (environmental pressure, cold water, long duration) (Chapin et al., 2021; Kelly et al., 2022a) is associated with hormonal imbalances, such as decreased testosterone, increased cortisol, and overtraining syndrome (Nindl et al., 2007; Kraemer et al., 2001; Nindl et al., 1985a). The hormone awake response provides insight into how the neuroendocrine system is responding to a heightened allostatic load, a common occurrence that arises during pre-deployment training (Khoury et al., 2015). Allostatic load is a term used to represent the physiological changes that occur because of repeated and chronic exposure to stress, which can result in a heightened neuroendocrine response and altered hormonal secretion (Taylor et al., 2016a; McEwen, 2015). Changes to the magnitude of such markers during the awakening process over time can give insight into the short-term and chronic effects of physical activity or training, and have been linked to overall health status (physical, physiological, and psychological) (Taylor et al., 2016a; Taylor et al., 2021; Taylor et al., 2016b). Critical to hormonal regulation monitoring is establishing baseline metrics prior to the onset of a heavy training cycle (e.g., pre-season, pre-deployment), in order to know whether an individual is reaching OTS or is at risk of reduced health or injury. Further, exercise stress, reduced energy intake, and poor sleep can lead to ar decreased sex-hormone production and increase stress hormones in male military personnel undergoing training (O'Leary et al., 2020; Szivak et al., 2018; Suurd Ralph et al., 2017; Nindl et al., 1985b). We recently reported that military personnel with high training volume had hormonal imbalances similar to those seen in OTS (Jensen et al., 2019). Hormonal regulation is coupled with metabolic load and energy intake, and thus understanding the metabolic demands of physically demanding military occupations is critical to maintaining readiness.

Military divers operate in high stress environments and to date, much of the research has focused on the medical aspects of diving (e.g., barotrauma, nitrogen narcosis, oxygen toxicity) rather than performance, which is vital for mission readiness and effectiveness, as well as health and longevity. Thus, the purpose of this effort was to quantify biomarkers of health, stress, and over-training prior to the annual training cycle, as well as characterize fitness and metabolic parameters of elite military divers. Population specific profiles will facilitate a more accurate physiological adaption of the impact of diving on military divers, as well as allow human performance specialists to customize diet and fitness programs.

## Methods

### Subjects

Seventy-eight active duty male elite military divers were included in the investigation. Participants’ data were aggregated over six training cycles occurring at various times of the year to ensure unbiased representation. Participants were informed about the potential benefits and risks associated with the study, and written informed consent was obtained prior to each participant’s involvement. This study was approved by the Institutional Review Board at Naval Health Research Center and adhered to the Department of the Navy human research protection policies (Protocol NHRC.2015.0022).

### Anthropometric measures

Body composition was obtained using Dual-Energy X-ray Absorptiometry (DXA) total body tissue quantitation (Lunar Prodigy, GE Healthcare, Madison, WI). Weight was measured using a calibrated digital scale to the nearest 0.01 kg (SECA, Germany). Height was measured to the nearest 0.01 cm using a stadiometer (SECA, Germany).

### Physical performance measures

To ascertain physical fitness, a modified Balke treadmill test (Khoury et al., 2015) was completed to assess each subject’s maximal volume of oxygen consumption (VO_2_ max). Subjects were instructed to warm up for 5–10 min by walking or jogging on the treadmill at a self-selected, comfortable pace. During the graded exercise test (GXT), the initial speed and grade of the treadmill were set at 3.5 mph (5.6 kph) and 0%. The speed and grade were increased by 0.5 mph (0.8kph) and 2%, respectively, every 2 min until volitional exhaustion, at which point the GXT was terminated. During the GXT, respiratory gases were continuously collected and analyzed using the Parvo Medics TrueOne 2400 (Parvo Medics, Inc., Sandy, UT, USA) metabolic cart system, and heart rate (HR) was measured using a Polar monitor (Polar Electro, Inc., Lake Success, NY, USA). Blood lactate concentrations were measured at the onset of the GXT, within the last 15 s of each 2-min stage, and at 2 min post-GXT. Capillary whole blood was collected via finger stick and used immediately to assess blood lactate concentration via a handheld lactate meter (Lactate Plus, Nova Biomedical, Waltham, MA, USA). Ratings of perceived exertion (visual 15-point Borg scale) were collected upon reaching volitional exhaustion (Taylor et al., 2016a). Successful achievement of VO_2_ peak was based on the following objective criteria: respiratory exchange ratio (RER), The ratio of carbon dioxide produced by the body to the amount of oxygen consumed ≥1.1, HR ± 10 bpm of age-predicted maximum, blood lactate concentration ≥8.0 mmol/L, and a rating of perceived exertion ≥18 on the Borg 15-point scale (McEwen, 2015). All the subjects met all the criteria during each GXT. For each subject, the ventilatory threshold (VT) was determined via the V-slope method as described (Taylor et al., 2021).

Measurements of strength and endurance were collected as part of their annual physical readiness test, which included a timed three-mile run, a timed half mile swim, pull-ups to momentary failure with 25 lbs (11.4 kg) attached at the waist, a standing broad jump, and an estimated 1 repetition maximum (RM) for bench press and dead lift via the Epley equation (LeSuer et al., 1997).

### Metabolic measurements

From the exercise stress test, metabolic data from indirect calorimetry were summarized into a digital data file and exported to Microsoft Excel. Absolute VO_2_ (L/min) and relative VO_2_ (ml/kg/min) were determined for 10, 25, 45, 65, and 85 percent of VO_2_max. Metabolic rate (kcal/min) at each intensity stage of VO_2_ max was determined by multiplying VO_2_ (L/min) at each stage by 5, the thermal equivalent of oxygen. Energy expenditure per hour was calculated by multiplying kcal/min by 60. Metabolic equivalent of task (METS) was calculated by dividing relative VO_2_ by 3.5. Macronutrient ranges by exercise intensity were calculated using current weight-based practice guidelines for active adults and tactical athletes from ACSM (Thomas et al., 2016), and ISSN position stands (Prado et al., 2022), where bodyweight was multiplied by the following: carbohydrate, 3–5 g/kg (light), 5–7 g/kg (moderate), 6–10 g/kg (high), 8–12 g/kg (very high); protein, 1.4–2.0 g/kg; fat, 0.8–1.5 g/kg. Total daily calories were then calculated using Atwater factors, multiplying grams of carbohydrate and protein by 4, and grams of fat by 9, then taking the sum to determine total daily calories.

### Salivary hormones

Participants self-collected salivary samples three times per day via passive drool in salivary collection tubes (Salimetrics, Inc., Carlsbad, CA), in a free-living environment. Standardized instructions for self-administration of samples were provided both in-person and in written format which outlined that samples be collected immediately upon waking (Wake), 30 min after awakening (Wake+30) and 60 min after awakening (Wake+60). All participants were encouraged to maintain their typical daily routines. Participants were required to record the exact time each sample was provided and any deviations from the testing protocol to help ensure compliance. Standardized instructions were given to the participants prior to data collection to ensure accuracy of the samples. Following collection of the saliva sample, participants were instructed to place each tube in their home freezer until retrieval of the samples, which were then transferred to a locked −80°C freezer until analysis. Data was collected at six time points throughout the annual training cycle.

Salivary cortisol, testosterone, and DHEA were analyzed in duplicate using a commercially available enzyme immunoassay kit (Salimetrics, Carlsbad, California). Cortisol (assay #1–3002) had a lower limit of sensitivity of <0.007 μg/dL and an assay range of 0.012–3.0 μg/dL; testosterone (assay #1–2402) had a lower limit of sensitivity of 1 pg/mL and an assay range of 6.1 pg/mL – 600 pg/mL; and DHEA (assay #1–1202) had a lower limit of sensitivity of 5 pg/mL and an assay range of 10.2 pg/mL – 1000 pg/mL. Summary parameters measuring the magnitude of the awake response included peak, area under the curve in respect to ground (AUCg), and average of the three wake-dependent cortisol measurements (Wake, Wake+30, Wake+60), and hormone reactivity (absolute and percentage) for each analyte were calculated as described by Taylor et al. (2016b).

### Statistical analyses

All data were analyzed using the statistical software R version 4.2.2 (R Core Team, 2022). Prior to any inferential analyses, raw salivary hormone data were screened for outliers. Summary parameters were calculated based on formulas used in previous analyses (Taylor et al., 2016). Deviations from normality in summary parameter data were treated with suitable transformations to bring skew and kurtosis values within an acceptable range of between −1 and 1, and -3 and 3 respectively. Descriptive summary statistics were calculated for all salivary summary parameters with means and standard deviations reported where appropriate. Overall subject level relationships were explored between all summary parameters and demographic/anthropometric, as well as physical performance metrics with Pearson product-moment correlations (low = 0.1–0.3, moderate = 0.3–0.5, high = 0.5–1.0). Where significant correlations were observed, a one-way analysis of variance (ANOVA) was performed on the quartile splits of each correlated metric to explore relationships further.

In addition to exploratory analyses performed across all six collection periods, a series of linear mixed effects models were fit to each summary parameter using the restricted maximum likelihood method to investigate the effect of training evolution date and time. In each model training collection time point was treated as a fixed effect while subjects were treated as random effects with varying intercepts. Significance of each model was calculated using Satterthwaite’s method to estimate degrees of freedom and generate p values. Variance contributions for fixed and random effects are estimated and reported as conditional and marginal R squared values (Nakagawa and Schielzeth, 2012).

## Results

### Participants

Seventy-eight elite male military divers from six different platoons located at the same command participated in this effort. Descriptive statistics of all anthropometric and performance measures are presented in Table 1. Results from the omnibus ANOVA revealed no statistical differences among platoons, therefore, all operators were combined for descriptive analysis. The individuals in this effort were between fit and highly fit based upon VO_2_max achieved (per ACSM values, between 50th percentile “good,” and 95th percentile “superior”) (Garber et al., 2011).

**TABLE 1:** Descriptive statistics for demographics.

Variable	n	Mean SD	Min	Max	IQR
Age (yrs)	77	28.0 ± 4.1	21.0	41.0	5.0
Height (cm)	77	179.7 ± 6.2	165.1	195.6	7.6
Weight (kg)	77	87.6± 8.1	70.3	106.6	11.3
Lean mass (kg)	70	70.5 ± 6.4	57.1	89.7	7.1
FFM (kg)	70	70.3 ±6.7	57.0	87.6	8.7
Fat mass (kg)	70	15.3 ± 5.2	5.1	29.6	7.7
Body fat %	70	18.1 ± 5.4	7.2	30.4	7.4
BMI (kg/m^2^)	77	27.1 ± 2.3	22.0	32.8	2.9
RMR - Mifflin St Jeor (kcal)	77	2144.0 ± 108.0	1918.0	2376.0	135.0
VO2 max (mL/kg/min)	74	55.0 ± 6.3	37.5	70.7	5.7
Blood lactate max (mmol/L)	73	13.7 ± 2.9	5.5	23.8	3.1
Lactate threshold (mL/kg/min)	73	47.2 ± 5.5	33.4	58.8	6.3
HR at lactate threshold (bpm)	72	180.0 ± 8.0	143.0	196.0	8.0
Ventilatory threshold (mL/kg/min)	73	49.0 ± 5.8	34.5	59.9	7.7
RHR (bpm)	63	76.0 ± 16.0	48.0	110.0	24.0
HR max (bpm)	75	192.0 ± 11.0	144.0	233.0	10.0
RER (VCO_2_/VO_2_)	74	1.2 ± 0.1	1.0	1.7	0.1
Weighted pull-ups (25lbs)	44	16.7 ± 6.4	7.0	40.0	7.3
Bench press 1RM (kg)	33	124.7 ± 19.9	94.2	172.2	22.6
Dead lift 1RM (kg)	37	181.6 ± 26.7	135.5	227.0	38.0
Broad jump (m)	45	2.4 ± 0.2	1.9	3.0	0.3
800-m swim (mm:ss)	33	12:51 ± 01:39	9:08	17:23	02:02
3-Mile run (mm:SS)	35	21:46 ± 01:59	17:23	25:45	03:02

Note: SD, standard deviation; IQR, interquartile range. 1RM, one repetition maximum.

### Metabolic metrics

From metabolic measurements, macronutrient estimates are presented in Table 2. All metrics were calculated using current weight-based practice guidelines from ACSM and ISSN position papers (Thomas et al., 2016; Prado et al., 2022): carbohydrate, 3–5 g/kg (light), 5–7 g/kg (moderate), 6–10 g/kg (high), 8–12 g/kg (very high); protein, 1.4–2.0 g/kg; fat, 0.8–1.5 g/kg. Total daily calories is a sum of each macronutrient in grams multiplied by their respective Atwater factor (carbohydrate: 4 kcal/G, protein: 4 kcal/G, fat: 9 kcal/G).

**TABLE 2:** Calculated daily macronutrient ranges and calories by intensity of exercise.

Exercise intensity	Carbohydrate (grams/day)	Protein (grams/day)	Fat (grams/day)	Total calories (kcal/day)
Light	263–438	123–175	70–131	2172–3635
Moderate	438–613	123–175	70–131	2873–4336
High	526–876	123–175	70–131	3221–5387
Very High	701–1051	123–175	70–131	3924–6088

### Salivary hormones

**FIGURE 1:**
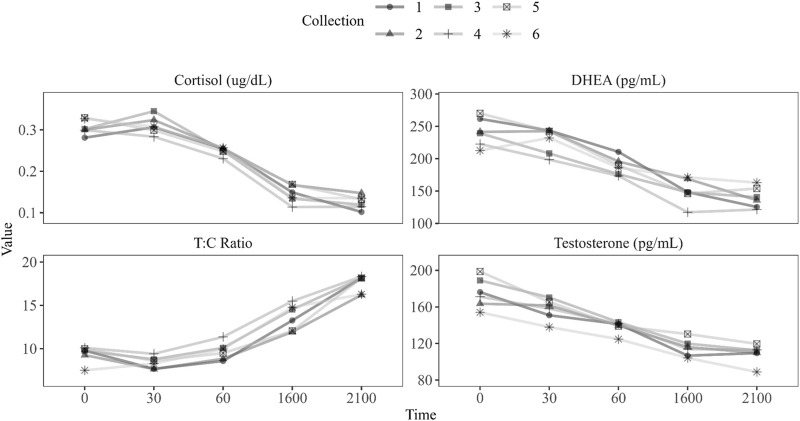
Hormone profile changes over time of day by ULT collection (1–6).

Descriptive statistics and summary parameters of salivary hormones are presented in Table 3. Hormone profile changes over the time of day are presented in Figure 1. Demographic variables showed no significant linear associations with summary parameters for both cortisol and T:C ratio when combined across all training evolution time points. Body fat percentage (AUCg: *r* (62) = 0.26, *p* = 0.038; Avg: *r* (63) = 0.3, *p* = 0.014; peak: *r* (63) = 0.31, *p* = 0.012) and fat mass (AUCg: *r* (62) = 0.25, *p* = 0.047; Avg: *r* (63) = .29, *p* = 0.019; peak: *r* (63) = 0.3, *p* = 0.014) both showed significant positive associations with all summary parameters of magnitude for testosterone, whereas blood lactate max (AUCg: *r* (63) = 0.33, *p* = 0.007; Avg: *r* (65) = 0.29, *p* = 0.019; peak: *r* (65) = 0.3, *p* = 0.015) showed significant positive associations with DHEA. Quartile comparisons for body fat percentage, fat mass, and blood lactate max yielded no significant differences. Further investigation into demographic relationships revealed no significant linear associations nor omnibus quartile differences for any other variables.

**TABLE 3:** Descriptive statistics for summary parameters combined across all 6 ULT training collection points.

	Cortisol (ug/dL)		DHEA (pg/mL)		Testosterone (pg/mL)		T:C ratio	
	n	Mean SD	n	Mean SD	n	Mean SD	n	Mean SD
Magnitude								
Peak	73	0.4 ± 0.2	73	280.7 ± 142.8	73	191.0 ± 72.6	73	12.1 ± 7.3
AUCg	73	17.6 ± 9.2	71	13384.3 ± 6482.8	72	9501.3 ± 3349.8	72	528.7 ± 281.8
Avg	73	0.3 ± 0.2	73	220.0 ± 109.0	73	158.0 ± 56.8	73	9.4 ± 5.5
Reactivity								
Absolute	73	0.01 ± 0.2	71	−14.0 ± 113.5	72	−19.0 ± 52.9	72	−1.1 ± 5.5
Relative (%)	73	13.9 ± 69.2	71	1.1 ± 47.0	72	−4.5 ± 36.9	72	−2.5 ± 48.5

Note: DHEA, dehydroepiandrosterone; T:C, testosterone to cortisol ratio; SD, standard deviation; AUCg, area under the curve with respect to ground; Avg, average.

Of the six physical performance tests completed by participants, all metrics but the 800-m swim showed significant relationships with at least one summary parameter. Significant negative associations were observed between relative cortisol and 25lb (11.4 kg) weighted pull-ups (*r* (38) = −0.42, *p* = .007) as well as between absolute DHEA and 1RM deadlift (*r* (32) = −0.4, *p* = 0.018). Significant positive associations were observed between AUCg testosterone and broad jump (*r* (39) = 0.31, *p* = 0.048); relative testosterone and three-mile run (4.8 km) (*r* (31) = 0.35, *p* = 0.047); as well as T:C ratio and 1RM bench press (Avg: *r* (29) = 0.37, *p* = 0.043; peak: *r* (29) = 0.39, *p* = 0.029), 1RM deadlift (AUCg: *r* (33) = 0.36, *p* = 0.033; Avg: *r* (34) = 0.44, *p* = 0.008; peak: *r* (34) = 0.39, *p* = 0.02), and 25lb (11.4 kg) weighted pull-ups (AUC: *r* (37) = 0.33, *p* = 0.038; relative: *r* (37) = 0.41, *p* = 0.01). Further comparisons in quartile splits revealed a significant effect of 1RM deadlift, long jump, and 25lb (11.4 kg) weighted pull-ups on absolute DHEA (*F* (3, 30) = 2.97, *p* = 0.048, *ηp* ^*2*^ = 0.23), AUCg testosterone (*F* (3, 37) = 2.86, *p* = 0.05, *ηp* ^*2*^ = 0.19), and AUCg T:C ratio (*F* (3, 35) = 4.66, *p* = 0.008, *ηp* ^*2*^ = 0.29) respectively. Bonferroni corrected post-hoc comparisons showed a significantly greater AUCg T:C ratio in the subjects who fell into the third quartile (15.6–20 repetitions) of 25lb (11.4 kg) weighted pull-ups (698.35 ± 266.06) compared to those who fell into the second quartile (12.76–15.5 repetitions, 426.82 ± 122.68, *p* = 0.031). There were no significant associations between quartile splits for 1RM deadlift and absolute DHEA, nor between long jump and AUCg testosterone when correcting for multiple comparisons.

**FIGURE 2:**
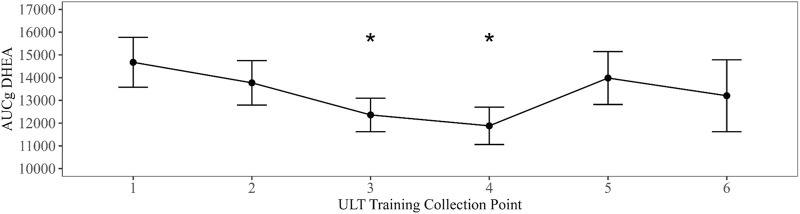
Changes in AUCg DHEA over ULT collection point (1–6). Asterisks represent significant differences from ULT training collection point 1.

**FIGURE 3:**
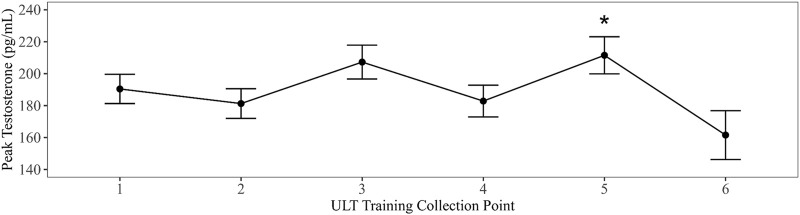
Changes in peak testosterone over ULT collection point (1–6). Asterisks represent significant differences from ULT training collection point 1.

Linear mixed models with training evolution collection time point as a fixed effect were compared to an intercept only null model in all summary parameters. Models that exhibited the best fit to the data revealed a significant main effect for training evolution on AUCg DHEA with collection time points three (B = −2735.96, *t* (177.32) = −2.39, *p* = 0.018) and four (B = −3089.92, *t* (178.97) = −2.7, *p* = 0.008) differing significantly from baseline (Figure 2). A significant main effect for training date on peak testosterone was also present in collection time point five (B = 28.12, *t* (215.4) = 2.4, *p* = 0.017) (Figure 3). Bonferroni corrected post-hoc comparisons showed significant differences between collections 2 and 5 (*p* = 0.029) and between collections 5 and 6 (*p* = 0.04) in peak testosterone. In both models, training evolution accounted for 3.3% and 4.1% of variance in the data for AUCg DHEA and peak testosterone respectively, with 38.34% and 38.77% of total variance coming from both fixed effects of training and random effects of individual participant differences. Adding training evolution time point as a fixed effect did not provide a more suitable fit in any of the other outcome variables.

## Discussion

Allostatic load refers to the cumulative impact of various stressors to include physical training, metabolism, as well as minor and major life events which interact and can challenge underlying physiology. Coupled to environmental challenges such as diving (undersea), overload of the physiological system can occur leading to pathophysiological disruption. Therefore, it is critical to understand at the simplest level, the impact of a training cycle on circadian rhythm as well as to characterize the individuals to determine physicality that may protective against the cumulative stress of training and environment. The data presented herein indicate there are certain periods of training that elicit significant changes in testosterone and DHEA while cortisol remains stable throughout the training cycle. To our knowledge, this effort is the first to document changes in stress biomarkers over time in elite military divers. Moreover, this effort comprehensively describes anthropometric, physical, and metabolic characteristics of elite divers and thus can serve as a reference for individuals that aim for a career as a military diver. Analysis of performance metrics reveal the individuals in the current effort have higher relative aerobic capacities when compared to the general military population (Sharp et al., 2008) and equivalent (Hunt et al., 2013; Muza et al., 1987) to other specialized units (Solberg et al., 2015; Sperlich et al., 2011). Few studies included parallel strength measurements, however, operators in the present study had greater upper body strength when compared to other elite international military groups (Solberg et al., 2015; Sporis et al., 2012). Furthermore, while elite military divers far exceeded body composition, aerobic performance, and strength requirements relative to current U.S. Navy standards, the results are consistent with previous research detailing various performance metrics in elite military populations (Nindl et al., 2007; Hunt et al., 2013; Muza et al., 1987; Solberg et al., 2015; Sperlich et al., 2011; Sporis et al., 2012; Dhahbi et al., 2016; Simpson et al., 2017).

Repeat salivary hormone profiles have been shown to be stable across two consecutive days in military cohorts (Taylor et al., 2016c) and that individuals with reduced DHEA and testosterone reported higher fatigue (Morgan et al., 2000). Others have reported changes in stress biomarkers over military survival training, which typically lasts for 3 weeks, and reported elevated cortisol with a reduction in testosterone with mock captivity. Here, we report that the average morning cortisol was less than what has been previously reported for elite military males (Hernández et al., 2021; Kelly et al., 2023). Further, there was a slight rise in cortisol after wake which follows a similar pattern as observed in other miliary populations (Taylor et al., 2016c; Hernández et al., 2021). Moreover, peak values are similar to those previously reported by our laboratory prior to a long duration dive (Kelly et al., 2022b). Changes in cortisol profiles over time can be indicative of overtraining, trauma and/or a habituation to a stressor (Pierce and Pritchard, 2016; Gerra et al., 2001). Coupled to cortisol release is secretion of DHEA. Both hormones are secreted in response to hypothalamic pituitary adrenal axis (HPA) activation and normally increase in response to physical and psychological stress (Kamin and Kertes, 2017). However, long term stress exposure (Lennartsson et al., 2013) and training stress has been shown to attenuate DHEA levels. Over the course of the training cycle, we see a decrease in DHEA between time points 1 to 4; however, DHEA recovers during the latter part of the training cycle.

Testosterone reached its peak at wake and steadily declined over the first hour of wake following its normal diurnal pattern. Absolute concentrations in this effort were like those previously reported in elite military men (Kelly et al., 2022b). However, over the course of training, testosterone levels decreased. Reduction in testosterone alone/or with elevated cortisol levels is a well-accepted marker of overtraining (Armstrong et al., 2021; Urhausen et al., 1995). Indeed, high cortisol levels have been correlated with low testosterone levels in over-trained athletes and high cortisol has been shown to inhibit testosterone production in healthy males (Carrard et al., 2022). Disruptions in the production of testosterone and cortisol have been implicated in physical and mental performance decrements (Urhausen et al., 1995) and the ratio between the two has been used to identify overtraining syndrome (OTS) in athletes. Chronic exposure to multifactorial stress, including training load and nutritional deficits, is associated with hormonal imbalances and may ultimately lead to OTS (Armstrong et al., 2021). Overtraining syndrome is generally characterized by reduced physical performance, increased fatigability, and subjective symptoms of stress coupled to low testosterone and high cortisol (Armstrong et al., 2021; Urhausen et al., 1995).

In the current effort, variations in cortisol awakening response (CAR) were measured but not statistically significant. Cortisol is diurnally regulated and in times of chronic stress can show a blunted awake response. We previously reported that 50% of military personnel tested during the training cycle had over-training syndrome and/or low testosterone (Jensen et al., 2019), necessitating a baseline assessment. In the current assessment, cortisol awakening response (CAR) had lower peak concentrations average concentrations (Figure 1) levels as compared to those previously reported (Taylor et al., 2016b). The blunted awakening response may suggest that the operators may be entering the training cycle with elevated levels of chronic stress (Duan et al., 2013). As theorized by Fries, Dettenborn (Fries et al., 2009), and Miller, Chen (Miller et al., 2007), a shift from hyperactivity towards hypoactivity of HPA axis develops continuous bouts of stress. Moreover, differences in the measures of magnitude may be a result of poor compliance during collection; however, this occurrence is unlikely as the specific measures of magnitude included in the study were chosen due to their robustness to the aforementioned issue (Taylor et al., 2016b). The awake response’s measures of magnitude (peak, average, and AUCg) have been shown to be stable across consecutive sampling days (Khoury et al., 2015; Pruessner et al., 2003) and robust to non-compliant subjects assuming the percentage of compliant individuals stands adequate (Taylor et al., 2016b). As subject compliance in other military populations (Taylor et al., 2016b) is comparable to other compliant populations in the clinical (Dockray et al., 2008) and general populace (Kudielka et al., 2003), it is likely adequate subject compliance was met and results are not driven by extraneous variables.

Much like cortisol, differences among the awake response of testosterone and DHEA were also seen. Consistent with previous literature, testosterone concentrations peaked upon waking and exhibited a decrease throughout the awakening response (Kraemer et al., 2001; Taylor et al., 2016a; Shariat et al., 2015). However, the magnitude of the anabolic analytes appears to be diminished when compared to other military in the literature (Taylor et al., 2016a). Decreases in testosterone and DHEA concentrations have been shown to stem from increased levels of physical and mental stress as shown in stressful work conditions (Chatterton and Dooley, 1999), U.S. Army Ranger training (Nindl et al., 2007), and individuals entering a state of overtraining (Urhausen et al., 1995). As these anabolic hormones can counteract common risks associated with the occupational stressors of the warfighter, maintaining adequate circulating concentrations is vital (Kessler et al., 2015; Harris et al., 2015). Analogous to cortisol, differences among the testosterone and DHEA awake response may be attributed to the day of collection (weekday vs weekend); however, to the authors knowledge, weekday to weekend alterations in the testosterone and DHEA awake response have yet to be investigated. Nevertheless, included results help provide a basis of the weekend awake response of testosterone and DHEA to ensure the hypothalamic pituitary adrenal gonad (HPG) axis is responding to training as expected.

There are some limitations to the current effort. The most prominent limitation was the subjects’ self-administered salivary samples rather than having oversight from the research team. Therefore, verification of the exact time of the samples to the Wake, Wake+30, and Wake+60 time-points was via self-report. Second, data on strength and conditioning metrics is incomplete as it is not a mandated assessment, and some individual’s scores were not gathered.

## Practical applications

Anthropometrics, physical, and physiological data help provide profiling information to help select adequate operators and provide a reference for when maladaptations to the training process occur. As mentioned by Maupin (Maupin et al., 2018), elite military personnel exhibit superior strength, endurance, and fitness capacities when compared to the general population or even general military. Therefore, the need to increase population specific research within the Special Operations divisions of the military will help focus the research efforts to the specific population. Furthermore, as evident by differences comparing literature within the same population, time specific comparisons must also be taken into consideration.

## Data Availability

The raw data supporting the conclusions of this article will be made available by the authors, without undue reservation.

## Author disclaimer

The views expressed in this article are those of the authors and do not necessarily reflect the official policy or position of the Department of the Navy, Department of Defense, nor the U.S. Government. I am a military service member or employee of the U.S. Government. This work was prepared as part of my official duties. Title 17, U.S.C. §105 provides that copyright protection under this title is not available for any work of the U.S. Government. Title 17, U.S.C. §101 defines a U.S. Government work as work prepared by a military service member or employee of the U.S. Government as part of that person’s official duties. Report No. 24–70 was supported by the Office of Naval Research under work unit no. N1506. The views expressed in this article are those of the authors and do not necessarily reflect the official policy or position of the Department of the Navy, Department of Defense, nor the U.S. Government. The study protocol was approved by the Naval Health Research Center Institutional Review Board in compliance with all applicable Federal regulations governing the protection of human subjects. Research data were derived from an approved Naval Health Research Center, Institutional Review Board protocol number NHRC.2015.0022.
